# Experiences of sexual relationships of young black women in an atmosphere of coercion

**DOI:** 10.1080/17290376.2013.807055

**Published:** 2013-06-18

**Authors:** Frances Clüver, Diane Elkonin, Charles Young

**Keywords:** coercion, female sexual health, HIV/AIDS, interpretative phenomenological analysis, qualitative, South Africa, santé sexuelle des femmes, VIH/SIDA, analyse phénoménologique interprétative, qualitative, Afrique du Sud, violence

## Abstract

Negotiations surrounding sexual activity are characterised by multiple power disparities that include race, social status and age, with gender being the most dominant differential in heterosexual interactions. Research has shown that women are physiologically more at risk of contracting HIV than men, as indicated by the higher infection rates of the former. Many African societies operate via a hegemonic masculinity, with patriarchal governance and female subordination being the norm, placing women at even greater risk of HIV infection. In this qualitative phenomenological study, four black school-going adolescent women living in Grahamstown were interviewed using a semi-structured interview to gather data. An interpretative phenomenological analysis was conducted on the data to provide subjective insights of the experiences of the participants with regard to their interactions with men. From the findings, it became apparent that the participants felt pressured, coerced or manipulated by male counterparts. This pressure and coercion was not just felt in their interactions with older men, but also in their romantic partnerships. Three of the participants experienced pressure to engage in sexual intercourse with their boyfriends when they were unwilling or unready, and they reported being faced with additional pressure to engage in unprotected sex. Furthermore, it became apparent that each participant had an underlying fear of being raped and considered this as a genuine threat to her safety and sexual health. The atmosphere within which these participants negotiate their sexual agency is thus heavily informed by male control, coercion and the threat of violence or rape.

## Abstract (FR)

Les transactions qui se font autour de l'activité sexuelle sont caractérisées par plusieurs jeux de force qui comprennent la race, le statut social et l'âge. Il est bien entendu ici que le genre est le facteur différentiel le plus important dans des relations hétérosexuelles. Des études ont montré que les femmes sont physiologiquement plus à risque de contracter le VIH que les hommes; comme leur taux élevé d'infection l'indique. De nombreuses sociétés Africaines fonctionnent à travers une hégémonie masculine, avec une gouvernance patriarcale et une allégeance féminine comme étant la règle. Ce qui expose les femmes à un risque encore plus grand d'être infectées par le VIH. Dans cette étude phénoménologique qualitative, quatre adolescentes résidentes de la ville de Grahamstown et élèves dans des ‘écoles pour noirs’, ont été interviewées en utilisant une interview de type semi-structurée pour collectionner des données. Une analyse phénoménologique interprétative a été conduite sur la base des données dans le but de fournir une compréhension subjective venant des participantes dans leurs rapports avec les hommes. Des résultats de recherche obtenus, il est devenu clair que les participantes se sentent sous pression, contraintes ou manipulées par leurs homologues masculins. Cette pression et contrainte n'étaient pas uniquement ressenties dans leurs rapports avec des hommes plus âgés, mais aussi dans leurs relations amoureuses. A titre d'exemple, trois des participantes ont été contraintes à avoir des rapports sexuels avec leurs petits amis alors qu'elles n'étaient pas consentantes ou prêtes. Elles ont également mentionné qu'en plus de cela, elles étaient forcées à avoir des rapports sexuels non protégés. En outre, il est devenu évident que chaque participante avait une peur radicale de se faire violer, et considérait cet acte comme une menace réelle pour sa sécurité et sa santé sexuelle. Ainsi, l'environnement dans lequel ces participantes négocient leur indépendance sexuelle est extrêmement dominé par le contrôle des hommes, la contrainte et la menace de la violence ou du viol.

Gender-related vulnerability has been recognised as a key aspect contributing to the increased propensity for women to contract HIV/AIDS, accounting for more women being infected than men (Macleod-Downes, Albertyn & Mayer 2008). Women are also physiologically more at risk with regard to contracting HIV than men (Harrison 2005). Moreover, in Africa, the female body is predominantly viewed as vile, debased and diseased (Abdool Karim 2005; Makahye 2005; O'Sullivan, Cooper-Serber, Kubeka & Harrison 2007). A study with young adults in South Africa revealed that both women and men viewed HIV as a ‘woman's disease’ (O'Sullivan *et al.* 2007). The women believed they had little power to avoid contracting HIV, while men were found to be of the opinion that their risk was linked to the illicit sexual behaviour of their female counterparts.

The sex act itself is the site of multiple power disparities that include race, social status and age, with gender being the most dominant differential in heterosexual interactions (Wood, Maforah & Jewkes 1998). Many African societies operate via a hegemonic masculinity, with patriarchal governance and female subordination being the norm (Fuller 2008; Naylor 2005). Women are cast in an inferior, dependent and passive role where their ideal qualities include submission, motherhood and ignorance, particularly about their sexuality and their bodies. In contrast to this, masculinity is depicted in terms of aggression, supremacy and autonomy, where their key merits are strength, valour and virility (Abdool Karim 2005). Harrison (2008) considers that it is important to be cognisant of cultural mores, values and beliefs in order to understand the specific sexual ideologies, relationship ideals and the construction and enactment of sexuality among members of diverse cultures.

## Social inequality and poverty

Female risk of contracting HIV is accentuated in South Africa by harsh social inequalities that exist between men and women (Fuller 2008; Varga 2001), while cultural norms have also been recognised as playing a central role in the spread of HIV (Castle & Kiggundu 2007; Frank, Esterhuizen, Jinabhai, Sullivan & Taylor 2008; Naylor 2005; Varga 2001). Numerous African cultures uphold traditional and religious practices that can, in contiguity with one another, aid in the spreading of this epidemic (Abdool Karim 2005; Fuller 2008; Macleod-Downes *et al.* 2008; Strebel, Crawford, Shefer, Cloete, Henda, Kaufman, *et al.* 2006; Thomas 2007). Patriarchal societal practices that subordinate the rights of women include cultural traditions such as lobola, polygamy and the economic dependence of women on men (Abdool Karim 2005; Fuller 2008; Macleod-Downes *et al.* 2008).

Women living in poor societies are at additional risk of HIV infection owing to the immediate needs for survival being placed above sexual health concerns (Abdool Karim 2005; Fuller 2008; Mathews 2005). Macleod-Downes *et al.* (2008) add that women living in lower socio-economic conditions are afforded less authority and social status, which further increases their risk of HIV infection. Thomas (2007) concurs that the two major factors that inhibit women's ability to protect themselves in relationships are their lower status in society and their financial dependence on men. Castle and Kiggundu's (2007) study with rural women in Limpopo reveals that these women lived in constant fear of HIV infection, but felt helpless and unwilling to leave their partners owing to economic benefits attached to being in a relationship. This situation increases the likelihood of sex being exchanged as a commodity, which, in turn, limits the decision-making ability of women and their ability to negotiate condom use, to refuse sex or to leave a promiscuous partner (Abdool Karim 2005; Harrison 2005; Macleod-Downes *et al.* 2008; Naylor 2005; O'Sullivan *et al.* 2007).

Cross-generational sex is characterised by young women having relationships with much older men, placing these women at risk of HIV infection for two reasons. First, such men often have a history of multiple relationships and are prone to coercive sex owing to it being exchanged for money and gifts (Frank *et al.* 2008; Fuller 2008) and, second, there is a power imbalance as younger women are unable to negotiate condom use or other conditions of sexual interaction (Fuller 2008; Harrison 2008). This type of transaction increases cross-generational infection rates and perpetuates the spread of HIV in younger women. Repercussions of this kind of relationship are serious as young women are initiated into a life where material possessions become accessible through prostitution, the danger of physical and sexual assault increases, and their health is compromised by the threat of HIV.

## Violence and coercion

Leach (2002) points out that other factors contributing to the spread of HIV are coercion, harassment and intimidation of women. According to the World Health Organisation, ‘violence against girls and women throughout the world causes more death and disability among women in the 15 to 44 age group than cancer, malaria, traffic accidents and even war’ (as cited in Naylor 2005:60). South Africa is said to have one of the highest rates of gender-based violence in the world (Jewkes, Sikweyiya, Morrell & Dunkle 2010; Seedat, Van Niekerk, Jewkes, Suffla & Ratele 2009). Gender-based violence can take many forms, yet it is usually sexual in nature and is often informed by the perpetrator's perception of socially acceptable forms of male and female behaviour, specifically stereotypical gender roles according to the individual's culture and context (Leach 2002).

Jewkes *et al.* (2010) and Macleod-Downes *et al.* (2008) discuss the fact that male dominance and sexual entitlement is central to understanding gender-based violence in South Africa, especially considering the country's accentuated gender hierarchy. They explain that the imbalance of power affords men the right, per se, to control sexual encounters entirely, including how and when sex takes place, and to use coercion in the form of emotional or physical pressure or even violence. Thus, coercion and violence could be considered to be by-products of the aforementioned patriarchal cultural traditions.

Naylor (2005) points out that when it comes to HIV prevention, strategies that advocate condom *negotiation* fail to recognise that equity between men and women in Africa rarely exists and that women in many cases do not have control or negotiation power in either consensual or coercive relationships. Similarly, Holland, Ramazanoglu, Scott, Sharpe and Thomson (1990) believe that effective public health campaigns aimed at women need to acknowledge that in the fight against STIs and HIV, women and men encounter their sexual experiences as unequal partners.

Leach (2002) reports that gender-based violence in African societies is rampant and extends into homes and schools. Wood and Jewkes (1998) conducted a study in two schools in the Eastern Cape in which they researched violence in adolescent sexual relationships. Their findings show that rape, physical assault and coercive sex were the norm. Masculinity was defined by the number of sexual partners a man had, as well as his ability to control his girlfriends – be it by force or threat. The young men in this study saw sex as their right and would use force to get it.

Within this complex matrix of social dynamics, women's agency and their power to negotiate are often undermined. Poverty and the subordinate position of women in African societies are the main factors that accentuate their reluctance to negotiate condom use and place them at risk of violence and coercion. Strebel *et al.* (2006) propose that successful and relevant interventions into sexual assault and gender relations first need to understand the complexities of the gender system in South Africa.

Violence and coercion in adolescent sexuality arenas, as well as the agency the female counterpart holds in such circumstances, have been neglected areas in health research and intervention development (Buga, Amoko & Ncayiyana 1996; Lesch & Kruger 2005; Wood *et al.* 1998). A limited amount of research on sexual relations in sub-Saharan Africa has been undertaken, with research on adolescents’ and young adults’ premarital relationships being particularly scarce (Meekers & Calvès 1997). Adolescents are developmentally at high risk of contracting HIV and of engaging in risky sexual behaviour (Ito, Kalyanaraman, Ford, Brown & Miller 2008; Mitchell & Smith 2003; Zisser & Francis 2006). Furthermore, sexual behaviours that emerge during this life stage – such as early sexual onset, multiple partners and infrequent condom use – can develop into habitual patterns that may jeopardise a person's sexual health in the future (Jones 2006). It is thus pertinent to look at personal accounts of coercion in intimate partnerships in South Africa, which are renowned for exceedingly high levels of interpersonal violence (Buga *et al.* 1996), so that more contextually based knowledge of how the phenomenon occurs and is experienced can be explored.

## Methodology

### Aim

The purpose of this study was to uncover the lived experiences of a sample of black school-going adolescent women with regard to coercion, manipulation and pressure in their sexual interactions with men.

### Research design

This qualitative exploratory study employs an interpretative phenomenological framework (IPA) to uncover the lived experiences of a small sample of adolescent women. IPA utilises an interpretative microanalysis of a small set of personal accounts so that the variability within human experience can be revealed (Eatough & Smith 2008; Smith 2004; Willig 2001) and, as such, is idiographic in nature, concentrating on the individual rather than the collective (Conroy 2003; Reid, Flowers & Larkin 2005).

### Sample

Purposive sampling from a volunteer focus group was used to obtain four black former Department of Education and Training school-going women between the ages of 16 and 17 who lived in Grahamstown in the Eastern Cape, South Africa. As a qualitative study, the sample neither strived to represent the population under study, nor aimed for generalisability, but rather it aimed to give rich, detailed accounts of the experiences of a few young black women in the Eastern Cape. This allowed for the exploration of the participants' accounts in a comprehensive manner so that their personal insights could be applied contextually to HIV/AIDS programmes and interventions.

IPA is appropriate to use with small sample sizes as it aims to illuminate detailed perceptions and understandings without the need to generalise the findings (Conroy 2003; Smith & Osborn 2003). Smith, Flowers and Larkin (2009) note that between four and six participants in a sample are sufficient for IPA research. IPA is concerned with the world as it is experienced by humans within their particular contexts (Willig 2001).

The educational context of the participants is known as a previously coloured school with a teaching medium of Afrikaans, but it now has a much larger black population (Lemon 2004). The school is situated in the coloured area known as Hlalani Village in Grahamstown and is poorly equipped and severely understaffed (Plaatjie 2011).

### Data collection

Before data collection commenced, ethical clearance was obtained through the Rhodes University Ethics Committee for Higher Education. Further, the Grahamstown Department of Education provided permission for the study to take place. The participants and their parents were fully informed of the nature of the study and signed consent forms.

The four participants were individually interviewed by the researcher in English as this is the medium of instruction at their school, as well as the common language between the researcher and participants. IPA already employs a double hermeneutic in that the researcher tries to make sense of how the participants make sense of their experiences. Thus, to avoid complicating the study unnecessarily, a translator was not used (Conroy 2003).

The interviews were semi-structured and an interview schedule was drawn up based on areas of concern in current literature. A few of the key questions explored during the interviews included: What happens in your family when subjects such as boyfriends, sex and HIV are brought up? Could you share with me any sexual experiences you have had? What were your thoughts and feelings when you experienced that? Do you use condoms and, if so, how do you negotiate this? Have you ever felt pressured into having sex and/or practicing unsafe sex? Have you ever felt manipulated or coerced into doing something you were uncomfortable with?

With the interviews being semi-structured, follow-up questions were asked where applicable and the participants were able to refuse to answer any of the questions. The participants' identities were protected via the use of pseudonyms – Gift, Kathi, Cindi and Tumi. Each interview was audio recorded, transcribed verbatim and thereafter erased.

The interviews were conducted outside school hours at the participants' school in a teacher's office, which was considered private, secure and neutral. Participants were provided with lunch and snacks as an incentive to take part in the study and this also contributed to a more relaxed interviewing atmosphere. Follow-up interviews took place to clarify and deepen initial results recorded and the participants were debriefed during a discussion group.

### Data analysis

An IPA was conducted on the raw data in this study, using the guidelines outlined in Smith *et al.* (2009) to provide subjective insights into the body of HIV/AIDS research. Yardley's (2000) guidelines for assessing the validity or trustworthiness of qualitative research were also utilised and adequately met. By using individual interviews to collect the data and IPA to analyse it, rich and deeply personal accounts were elicited, offering the different participants' perspectives. The interactive and inductive analysis as outlined by Smith *et al.* (2009) was followed carefully.

## Findings

During their interviews, the participants highlighted experiences in which they had felt pressured, coerced or manipulated by their male counterparts. Outside their romantic relationships, the participants had felt coerced by strangers in taverns, and, in one instance, by a male teacher. Weiss, Whelan and Gupta (2000) believe that sexual coercion and violence are pervasive in adolescent relationships, yet are often covert as they happen within a very private sphere. When the participants revealed their sexual experiences, it became apparent that three of them had encountered pressure to engage in sexual intercourse with their boyfriends when unwilling or unready. Although it is commonly anticipated that sex is part of the relationship contract, the expectation and act thereof were effected coercively. Once sexually active, the participants disclosed that male control was additionally exerted, albeit unsuccessfully, as a means to initiate unprotected sex.

Furthermore, it was evident that each participant had an underlying fear of being raped and considered this a genuine threat to her safety and sexual health. The atmosphere within which these participants negotiate their sexual agency is thus heavily informed by male control, coercion and the threat of violence or rape.

### Encounters with older men

Commonly, younger members of society have less power than older members and it is younger women who have significantly less power than men of any age in South Africa (Abdool Karim 2005). It is thus not surprising that the participants in this study reported being mistreated and coerced by older men in taverns. Although they appeared to have handled these pressured situations well, it seems unsettling that they should have to ward off the coercive advances of older men on evenings out with their friends. Both Tumi and Cindi commented that men in taverns frequently buy women drinks in the hope that this will lead to sex. Cindi explained one of her experiences:

He came to me and greeted and I said, ‘Hello, dad’ and he asked me why I am saying dad to him and I told him that he's even older than my father so I was trying to respect him. He said that no, he's not old, it's just his body …. Then he told me, ‘What can I buy you?’ and I said, ‘No, I didn't come to you I just came to have fun so just leave me alone.’ And then he said, ‘You think you are clever. You think you are sexy’ then he called me a bitch.

The fact that Cindi was being offered a drink by a much older man revealed to her his unvoiced intention to have sex with her. Cindi's reluctance to accept the drink (and therefore the sex) made him angry and provoked him to insult her. This form of verbal abuse or intimidation does not appear to happen in isolated incidents: indeed, participants mentioned it was a regular occurrence.

Tumi explained how men treated her and her friends when they refused to accept alcohol from them:

And then they [a few men] were sitting with us and the other guy was holding a glass and he passed it to my friend and she refused it so then they were like, ‘No, you think you are better than us’ and we were like teasing and we were laughing …. They do get cross but we just laugh. And if they say, ‘We're going to hit you’ we just tell them we're going to run or that we're going to cry ‘cause it's going to be painful and then that's it’.

Tumi voiced the above statement light-heartedly, implying that coercion and the threat of physical violence is neither surprising nor unexpected. The above instances expose the atmosphere in which the participants in this study socially interact and negotiate with men. The coercive nature with which these men engage with the adolescent participants shows a disregard for women and their rights, and reinforces a patriarchal system of dominance.

Gift shared the story of an evening when a male teacher at her school offered her a lift home and pressured her to engage with him sexually. He drove to a secluded location in Grahamstown and when he tried to kiss her, she began to defend herself against his advances. She shared the following:

So I was like, ‘Dude, you're my teacher, you're not supposed to do this stuff’ and he was like, ‘I know but you're so hot, I like you, I really like you.’ And he was really holding me and stuff. So I asked him to please take me home ‘cause I'm really uncomfortable with this and he was like,’ ‘You don't have to be uncomfortable, I mean, you know me.’ … and he started kissing me and all that stuff and I was trying to tell him to stop doing this ‘cause I really didn't want to do this and I was uncomfortable and all that.’ But he was definitely not listening to me because he just carried on … and then he was undressing himself and I was like, ‘Dude, really, I don't want to do this I just want to go home.’ … So ja, he was getting really undressed and saying things like, ‘You really turn me on’ and ‘Feel my thing’ yadda yadda. And I was like, ‘No, I don't want to feel your thing’ and he told me, ‘You're really not listening’ [forceful tone used].

Gift was able to stop him from raping her and fended him off by asking for more time to think about sleeping with him. After her teacher dropped her at home, Gift felt a whirlwind of emotions, as she described below:

I was really, really scared. I felt dirty. I was confused. I was shocked – I didn't think a dude was capable of doing that. So when it happened I kind of felt numb.

It is evident from the above extracts that Cindi, Tumi and Gift all experienced coercion and intimidation in their interactions with older men.

### Pressure to have sex within romantic relationships

Kathi explained how her boyfriend brought up the subject of sex so often that she felt she had to tell him to find another girlfriend if he was not happy to wait until she was ready. The process of becoming ready for sex was not elucidated by the participants, but utilising the phrase in this context suggests Kathi used it as a temporary means to deflect her boyfriend's constant demands for sex. However, she considered sex to be an inherent and inevitable part of the romantic partnership. This, together with her boyfriend's expectation of having sex, possibly prompted Kathi to feel it necessary to ready herself for it. Once Kathi became sexually active with her boyfriend, he seemed to expect that they would have sex whenever he wanted to thereafter. She explained that the second time she had sex with her boyfriend, she was not expecting it and felt dirty as a result:

I expected to be going there to chat with him and get to know him a little bit more. But when we engaged into having sex, I didn't feel very comfortable with it and I never told him that ‘cause I didn't want to make him feel bad …. I didn't really expect it but he's thinking that I'm used to him now and we had sex already and he thought it was fine for us to engage into sex again but he didn't know that I wasn't really into sex …. Afterwards you know, when somebody is raped they feel very dirty. I could say I did feel like that because I didn't expect to have sex that day. So I felt a bit dirty’.

This excerpt reveals that Kathi's boyfriend had power over her when they engaged sexually and this limited her ability to be honest about her needs. She may also have felt the pressure to satisfy her boyfriend constantly, in order to maintain the relationship and to prevent him from seeking sexual gratification elsewhere. These findings further verify those of Abdool Karin (2005) and Macleod-Downes *et al.* (2008). Jewkes *et al.* (2010) concur that many women believe that they are legitimately unable to refuse sex with a husband or boyfriend, to the degree that sexual coercion by an intimate partner is often not regarded as being rape (Jewkes *et al.* 2010).

At the end of the above extract, Kathi claimed she felt ‘dirty’. She used the word ‘dirty’ to bring to our attention that she was being used to satisfy somebody else's sexual needs – perhaps she felt prostituted. Harrison (2008) explained that the word ‘dirty’ has connections with the moral implications of having sex before marriage and with sex being generally constructed as bad. Kathi also likened her emotional experience of the event to that of being raped. She used the metaphor of rape with its associated feelings as a way to illustrate that she neither wanted nor enjoyed having sex that day. At the same time, she felt emotionally unprepared and was unable to voice her unwillingness.

Tumi shared that her boyfriend manipulated her into having sex with him and, owing to her young age at the time, she capitulated. She expressed the following thoughts:

Okay, well I think I broke my virginity when I was about fourteen because I think it was lack of knowledge at first. I had a boyfriend who was around about eighteen years I think. So it was like, more pressure on me …. He knew I was a virgin and he kept asking me when we were going to have sex, but I was like, ‘But I'm not ready’ and he said, ‘There's no need to be scared, there's nothing to be scared about, it's fine. There's nothing painful. I will take care of you.’ I said, ‘No, no, no. I don't want’ and he kept on going. And you know, boys are very impressive and sometimes he said impressive things like, ‘I love you’ and stuff the whole time, you know? Then it just happened.

Tumi's words reveal the magnitude of the consistent pressure her boyfriend placed on her to sleep with him. She stated that she had told him clearly that she was not ready and that she did not want to have sex but ‘he kept on going’. In the interview, she admitted being somewhat ambivalent about wanting to have sex in the first place and that had she not faced so much pressure, she would not have succumbed.

The third participant to experience pressure to have sex was Cindi. She spoke of an occasion when her boyfriend woke her late at night and was forceful with her because he wanted to have sex. She revealed:

So I was sleeping and then I felt someone touching me and then I thought he was sleeping but he was awake and he wanted to have sex. I told him, ‘No, I'm not in the mood of having sex’ and then he told me, ‘Come on’ [forceful tone used] and then he told me that [laughs] I'm being moody and then I said that I wasn't being moody and he said it was like I'm changing and I told him, ‘No, I'm not changing but I'm not in the mood.’ … so he wanted to know what was the secret behind. I told him, ‘No, I don't have any secret behind I just don't feel like it’ and then he was angry …. Then he apologised and I said, ‘No problem’ and he never did it again. So from that day he never drank again.

In this incident, Cindi was successfully able to refuse sex with her boyfriend, with whom she was already sexually active. However, she was met with accusations of hiding information, which her boyfriend assumed to be the reason why she was unwilling to engage in sexual intercourse. She blamed his forcefulness and anger during this confrontation entirely on the fact that he had been drinking that night. Jewkes *et al.* (2010) found that although alcohol in itself does not cause men to rape, strong evidence suggests that alcohol is part of the context of many acts of rape.

Refusal by a woman to submit to the sexual demands of her partner is said to signify to a man that she has other sexual partners and has thus been ‘worn out’ (Wood *et al.* 1998:238). Certain groups of Xhosa adolescents in the Eastern and Western Cape deem this true and it could be applicable to the participants in this study. Perhaps Cindi's boyfriend had this very fear when he accused her of hiding something from him.

Consistent with these results, Abdool Karim (2005) found that many young women in South Africa are coerced into having sex through gender role expectations about love, sex and compliance with a man's desires. Further, owing to the imbalance of power in favour of men, there is their perceived right to control sexual encounters, including how and when sex takes place and the use of coercion in the form of emotional or physical pressure (Macleod-Downes *et al.* 2008). In their sample of Xhosa adolescents in a township in the Western Cape, Wood *et al.* (1998) found that the conditions and timing of sexual intercourse were delineated by men. This was often exerted through the use of violent behaviour, as well as through perpetuation of certain constructions of love, entitlement and intercourse to which the young woman were expected to submit. Similarly, among their sample of adolescent Xhosa women, Buga *et al.* (1996) reported that violence, or the threat thereof, was a consistent feature of their sexual relationships and was used to enforce male control of sexual intercourse. The participants' accounts in this study reveal that sex is mostly initiated by the male partners through the use of pressure, coercion or manipulation.

### Pressure to have unprotected sex

The use of condoms and the negotiation thereof was another area in which the participants encountered pressure from their boyfriends. Although they were able to insist on consistent condom use, the participants revealed they faced constant demands for unprotected sex.

During Tumi's first sexual encounter, her boyfriend used the myth that virgins cannot get pregnant when they have sex for the first time. Apparent here is the fact that some men will lie, perpetuate myths and exploit a girlfriend's naivety in order to have unprotected sex. Kathi's boyfriend, on the other hand, chose to assure her of his negative HIV status to calm her fears, hoping that she might feel safe enough to sleep with him without protection. She recalled:

He told me one time to try it without a condom and I told him, ‘I won't do that because I've explained to you in the past that I do not want to have sex without a condom due to HIV and AIDS risk you know.’ And then he told me, ‘But I don't have HIV’ so I said, ‘I don't know about that because I haven't seen a paper, written in black and white that you don't have HIV and the fact that you were with the other girls, like you've had previous sexual relationships – I don't know what happened in those relationships so I don't know if those girls are HIV or maybe you have a sexually transmitted disease. And I can't go ahead with that until I know for certain that I am safe and I'm ready to have sex without a condom’.

Kathi's words ‘I've explained to you in the past’ reveal that this was not the first time she and her boyfriend had had this conversation. She claimed her fear of disease was the main reason she did not want to engage in unprotected sex, despite his efforts to prove that infection was not an issue.

In the following extract, Gift explains her boyfriend's demands that she had birth control injections so that they could have unprotected sex:

… he was saying that this protection thing was really not comfortable for him and he asked me if I can go to the clinic and all that stuff so that we didn't have to use protection.

Gift's boyfriend used his experience of pleasure (or lack thereof owing to condom use) as a means of manipulating her into using hormonal contraceptives so that he could have unprotected and more pleasurable sex. This is not a unique occurrence. Other research has shown that men generally prefer the experience of unprotected sex (Makahye 2005) and have been known to pressure their partners to be on alternative birth control so that they can achieve this (Harrison 2005; Naylor 2005; O'Sullivan *et al.* 2007).

### Living with a constant fear of rape

It became apparent during the interviews that each participant had an underlying fear of being raped and considered rape to be a genuine threat to her safety and sexual health. When talking about her decision to lose her virginity, Gift said:

I mean, rape has always been one of my fears since I was like twelve. Ja, so I was like, okay you know what, my first time instead of it being all traumatic, let me just do it now and get it over with.

Gift's decision to have sex for the first time was based on her fear of being raped as a virgin. She expanded on her reasoning thus:

Now I thought, you know what, being raped is being forced and all that stuff so I thought I don't want my first time to be like that because it says somewhere on TV or I don't know where, that if it's your first time and you're being raped it will be traumatic like for the rest of your life. So yeah, I decided just to do it [have sex with her current boyfriend].

It can be surmised that Gift's fear of rape drove her to become sexually active so that if she was raped thereafter, it would be less traumatic. This seems an extreme measure for Gift to take in order to protect herself. It also shows how real and immediate Gift's fear of rape was and how it pushed her to take some form of control over her body by actively deciding to have sex.

From a young age, Kathi also feared the prospect of being raped. The following statement shows how she considered it a potential negative outcome of an otherwise innocent school crush:

Well a boy in primary school had a crush on me and I didn't know but he came to me and he kissed me and at first I was scared ‘cause of rape and all of that. I thought maybe he wanted to rape me but I found out later on that he only had a crush on me’.

The association Kathi made between someone being attracted to her and that person having the intention of raping her is alarming. However, it seems less surprising when one considers that the participants' context was self-evaluated as dangerous and conducive to rape.

Kathi mentioned witnessing a young woman being forcefully taken into a dark alley when walking home from school one evening. Cindi and Tumi shared instances where they saw men taking reluctant women into their cars or behind taverns on evenings out. Furthermore, Gift shared that her fear of rape stemmed from her friend's experience thereof. This suggests that rape is not an external threat, but rather that the participants had witnessed it or been in direct contact with someone who had been raped.

When asked whether they feared being raped, Cindi and Tumi responded that they had not thought about the prospect directly. Rather, they negotiated the prospect of rape in terms of danger management. Below, Cindi explains what she does in order to avoid being raped:

Uh, I first of all avoid being in the company of drunk people and drugged people – people who are using drugs – and people that I can see would like to take advantages and I avoid being in a group of friends ‘cause sometimes friends can be dangerous. So I prefer not to have a friend. I don't have friends actually, I only have my boyfriend as my friend’.

Cindi acknowledged that substance abuse heightened the risk of potential rape situations and thus she avoided being in the company of such people to protect herself from becoming a sexual target. She also noted that friends could be dangerous and commented that she kept her boyfriend's company as a tool by means of which she defended herself and kept herself safe.

Initially in the interview, Tumi did not regard rape as an immediate or conscious threat. Through her response, however, it is evident that rape was an intrinsic danger that warranted personal concern:

Yo … I think for me I've never thought about like one day I'm going to get raped, you know …. Like for instance, you must avoid things that you like, will lead you in that way …. In your home if you don't trust your uncle, if you feel something in your heart you say: you know what, I'm not comfortable it's better to move away or go to my friend's house. And the other thing: never walk at night alone after five o'clock even at home they don't allow me to walk alone, you know? And these people go out drinking at night and they come back then they're walking alone you know …. Alcohol sometimes leads to rape. I think for for for people who are like more than sixteen years, you know, they should avoid that but the for children aye, it's another thing because it just happens in their families but for other people they should just avoid roads that lead to rape.

In the above accounts, it is interesting to see that although Cindi and Tumi did not conceptualise rape as an immediate threat, they intuitively formulated ways to avoid it. For Cindi, it was avoiding people who are under the influence of alcohol or drugs and for Tumi, it was a matter of conducting herself in ways that circumvent ‘roads that lead to rape’.

These findings reveal that the participants experienced coercion and pressure, both in their interactions with older men and within their romantic relationships; that they faced pressure to have unprotected sex; and negotiated all this pressure while experiencing an underlying fear of being raped.

## Limitations and suggestions for future research

A limitation in this study is that the researcher is an older, white female interviewing younger and less economically privileged black adolescents and this power dynamic may have influenced their responses. Historically in South Africa, it is claimed that white women have been speaking for black women and white middle-class voices dominate in knowledge production when it comes to research (Lesch & Kruger 2005). The researcher remained cognisant of this dynamic.

The participants voiced their experiences to someone outside their social context and, while this may have been a good opportunity to inform outsiders of their lived realities, this may have limited their ability to go into detail. Thus, the participants may have focused more on painting the broader brushstrokes of their socio-cultural pictures than particular details of their lived experiences. Being an outsider meant that the researcher could only interpret the participants' expressions from her own position. This may have limited her ability to immerse herself completely in the understandings and meanings attached to their experiences and realities. Having said this, it may have been beneficial to have an outsider in that the participants were not limited by the perceived expectations of those in their communities. This may have consequently led to more honest accounts.

An inability to generalise the findings owing to the small sample may be a weakness in this study, but IPA esteems idiography above generalisability (Smith *et al.* 2009) and a detailed analysis was conducted on each of the four interviews. This allowed for the exploration of a few participants' accounts in a meaningful and comprehensive manner, so that their personal insights can be applied contextually to HIV/AIDS programmes and interventions.

In the field of HIV research, it is important to look at sexual activity, situations and behaviours within their social contexts (Flowers, Marriott & Hart 2000). With this in mind, more interviews with a larger sample may have fleshed out some of the commonalities and differences in the accounts. Future studies could involve female participants of different races, from diverse backgrounds and from former Model-C schools as well as private schools. In this way, the salience of themes that emerge in the participants' accounts and any notable discrepancies could be assessed.

Future research could also explore the experiences of a sample of young black men with regard to aspects of power and coercion in their romantic relationships. Such research would complement studies that focus on women and their vulnerability to HIV. There has been growing recognition of the need to involve men in sexual health initiatives, but it has been noted that male health issues in terms of HIV/AIDS have been eclipsed by an emphasis on female concerns (Varga 2001). Men are equally, perhaps more, inclined than women to behave in sexually risky ways (Koster, Kemp & Offei 2001), which correspond with gender roles and ideals, as well as notions and expressions of masculinity (Varga 2001). Koster *et al.* (2001) suggest that HIV/AIDS intervention programmes designed for young men have been lacking, if not completely absent. This points towards the need for gender-based and context-specific research that can supply empirical and educational insight and material for young men.

As has been noted with regard to women and HIV, descriptive and exploratory research on adolescent sexuality and sexual behaviour in diverse South African communities is needed urgently. It is clear that certain groups of low-income women in South Africa have been largely neglected in terms of research in the field of sexual agency (Lesch & Kruger 2005).

## Conclusion

The findings presented here describe the coercive sexual dynamics operating within the participants' lifeworlds. Coercion occurs not just in taverns with strangers, but also within the participants' ongoing romantic partnerships. Three of the participants reported that they had been manipulated or coerced into sex. Control was additionally exerted as a means of initiating unprotected sex. Male control and coercion was also seen in terms of the threat of rape evident in the participants' accounts.

These data endorse the necessity of obtaining in-depth understanding of sexual experiences to shed light on sexual decision-making within an atmosphere of pressure, coercion and manipulation. Wood *et al.* (1998) propose that sexual health initiatives be mindful of the power differentials at play in heterosexual relationships. Such dynamics can manifest in violent and coercive practices that limit young women's ability to negotiate and exercise their agency to protect themselves against undesired sexual intercourse, HIV, STIs and unwanted pregnancy.

It has been established that the HIV/AIDS epidemic is an area of crisis amongst South African women (Abdool Karim 2005; Castle & Kiggundu 2007; Fuller 2008; Harrison 2005, 2008; Leach 2002; Lesch & Kruger 2004; Macleod-Downes *et al.* 2008; Thomas 2007; Wood *et al.* 1998). This crisis needs to be addressed not merely through the publication of comprehensive research, but via relevant intervention strategies at the grassroots level. Wood *et al.* (1998) suggest that this is possible through the initial use of localised qualitative research. Such research serves to give participants a voice and to contextualise the individual's dynamic lifeworld within social settings. This will in turn contribute significantly to the development of locally appropriate, as well as culturally and contextually sensitive, sexual-health interventions.
